# Synbiotics are Better Than Probiotics and Prebiotics for Use as Nutrition in Growing Rabbits

**DOI:** 10.1002/vms3.70977

**Published:** 2026-05-14

**Authors:** Khalaf Alrajab, Taghi Ghoorchi, Abdolhakim Toghdory, Omid Ashayerizade, Farzaneh Ganji

**Affiliations:** ^1^ Department of Animal and Poultry Nutrition, Faculty of Animal Science Gorgan University of Agricultural Sciences and Natural Resources Gorgan Iran; ^2^ Department of Animal Production, Faculty of Agriculture Engraining University of Aleppo Aleppo Syria; ^3^ Department of Biology, Faculty of Science Golestan University Gorgan Iran

**Keywords:** blood parameters, digestibility, growth performance, prebiotic, probiotic, rabbit, synbiotic

## Abstract

**Objective:**

This study evaluated the effects of probiotic, prebiotic and synbiotic supplements—specifically Saccharomyces cerevisiae and Mannan‐Oligosaccharide (MOS)—on growth performance, nutrient digestibility and blood parameters in growing rabbits.

**Materials and Methods:**

A total of 32 weaned rabbits (56 days old; 450 ± 50 g) were randomly assigned to four treatments (eight replicates each) in a completely randomized design. Housing facilities were disinfected before the trial, and rabbits were treated with ivermectin to control internal parasites. The experimental diets included a basal diet (control), a basal diet with probiotic Biodep (0.02%), a basal diet with prebiotic Agrimos (MOS) (0.2%) and a synbiotic combination of both additives. The experiment lasted 56 days. Body weight (BW) was recorded biweekly to determine growth performance indicators, including BW, average daily gain (ADG), dry matter intake (DMI) and feed conversion ratio (FCR). At the end of the trial, blood samples from four rabbits per treatment were analyzed for glucose, cholesterol, triglycerides, lipoproteins (HDL, LDL and VLDL), white blood cells and red blood cell indices. Skeletal growth and intestinal morphology were also assessed.

**Conclusion:**

The synbiotic group showed the best growth performance and feed conversion efficiency. Nutrient digestibility, including dry matter, organic matter, crude fat and fibre (NDF), was significantly improved. Blood analysis revealed significantly lower cholesterol and immune cell counts in the synbiotic group (*p* < 0.05). Skeletal growth was also enhanced, with significant improvements in most measurements. Overall, synbiotics demonstrated substantial benefits and represent a promising nutritional strategy for improving rabbit production.

## Graphical abstract

This study demonstrates that synbiotic significantly enhances growth performance and nutrient digestibility in growing rabbits. The synbiotic approach improves feed conversion and skeletal development while lowering serum cholesterol and modulating immune cell counts. These findings highlight synbiotics as a potent nutritional strategy to optimize rabbit production through enhanced gut health.

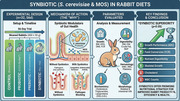

## 1 Introduction

In both developed and developing countries, the consumption of rabbit meat is steadily increasing worldwide. Rabbits are categorized as intermediate between ruminants and monogastric animals, as they can efficiently utilize cellulose‐rich feed when diets contain less than 20% grain (Fraga 1998). They possess a short reproductive cycle, high fertility and excellent feed conversion efficiency, which places them just below chickens in productivity rankings (Hasanat et al. 2006). The gut microbiota of rabbits plays a vital role in maintaining their health and facilitating digestion. Proper management of this microbiota is essential, as a balanced gut microbiota significantly contributes to rabbit performance and well‐being. However, rabbits are highly susceptible to pathogenic microflora, which poses a significant biological risk to their husbandry (Kurchaeva et al. 2020). Because of their high feed efficiency, quick growth rates and capacity to adapt to a variety of farming systems, rabbits have become an essential part of sustainable meat production (Ashour et al. 2024; Abou‐Kassem et al. 2025). Rabbit meat is considered one of the healthiest meats due to its beneficial nutrients and properties, such as low fat, low sensitivity, low cholesterol, high digestibility and a significant amount of unsaturated fatty acids (Dalle Zotte et al. 2016). It is classified as a functional food because of its high digestibility, essential amino acids, unsaturated fatty acids and its rich content of phosphorus and calcium. Furthermore, rabbit meat contains lower levels of fat, cholesterol and sodium compared to traditional red meats (Ma et al.^2022^). These nutritional qualities have contributed to its growing popularity on a global scale (Krunt et al. 2022). Also, Khan et al. (2025) investigated the phytochemical composition and synbiotic relationship of fruit peel powder (FPP). The FPPs showed variations in moisture, ash, protein, fibre, fat and carbohydrate content.

Among various yeast species, *Saccharomyces cerevisiae* (*S. cerevisiae*) is extensively used as a probiotic supplement in animal production. This is due to its rich composition, which includes proteins, polysaccharides, small peptides, amino acids, vitamins, trace elements, nucleotides and other growth factors (Pang et al. 2022). Consequently, it has been reported that *S. cerevisiae* enhances animal production performance, strengthens immune function, regulates the balance of intestinal flora, promotes intestinal development and improves meat quality (Bin et al. 2019; Elghandour et al. 2020). In this regard, probiotics, live microorganisms that help the host, have become popular substitutes for antibiotics, improving gut health, growth and immunity in animals with monogastric (Cufadar et al. 2024). Probiotics containing live bacteria, bacterial spores, or yeast can help inhibit rabbit gastrointestinal disorders. According to Forgie et al. (2019), it may involve lowering the production of toxins, boosting the host's enzymes, producing certain germicide compounds, competing for sticking to epithelial cells, boosting the immunological response of the host and lowering stress levels in rabbits. Furthermore, by lowering gut pH, probiotics may decrease the number of dangerous bacteria (Youssef et al. 2024). Numerous additional boosted effects of probiotics were proposed, including lowered inflammatory responses, reduced excretion of urea and ammonia, decreased serum cholesterol and enhanced mineral absorption. Additionally, probiotics might indirectly improve production profitability and performance metrics (Jha et al. 2020; Mousa et al. 2022). On the other hand, prebiotics are indigestible carbohydrates that can be fed to animals to modulate the balance and activity of microbial populations in the gut (Ma et al. 2022). Mannan‐oligosaccharide (MOS) and β‐glucan, derived from the cell wall of *S. cerevisiae*, are notable examples of prebiotics. These innovative feed additives have demonstrated biological functions, such as supporting the development of the animal gastrointestinal tract, regulating intestinal flora, enhancing immunity, improving feed efficiency and boosting overall growth performance (Kango et al. 2022). Synbiotics refer to a carefully designed combination of probiotics and prebiotics, intended to support the growth of probiotics by supplying specific nutrients for fermentation within the gastrointestinal tract (Dankowiakowska et al. 2019). These combinations offer significant advantages to the host by improving the survival and presence of beneficial microbial supplements in the digestive system (Elliethy et al. 2022).

Abdelhady and El‐Abasy (2015) in their study referred to the effect of prebiotics and probiotics on growth, blood immune responses and blood parameters of rabbits infected with *Pasteurella multocida*. They found that the effect of prebiotics and probiotics and their mixtures on healthy or *P. multocida*‐infected rabbits improved growth, cellular immune responses and blood and serum biochemical parameters, reduced mortality and improved adverse clinical signs and post‐mortem lesions. Moreover, rabbits are unable to produce cellulolytic enzymes on their own and, therefore, rely heavily on gut bacteria for the digestion and utilization of dietary fibre (B. Liu et al. 2022). The combination of probiotics and prebiotics presents considerable potential in rabbit production. It can improve feed efficiency, growth performance and overall health by promoting the competitive exclusion of harmful microorganisms and exhibiting antimicrobial properties. However, limited information exists regarding the combined effects of these supplements in rabbits. Consequently, this study aims to evaluate the impacts of probiotic, prebiotic and synbiotic feed supplements specifically incorporating the commercially available probiotic *S. cerevisiae* and MOS prebiotics on performance, digestibility and blood parameters in growing rabbits.

## 2 Materials and Methods

### 2.1 Experimental Animals and Diets

This research was conducted at the educational and research farm of Gorgan University of Agricultural Sciences and Natural Resources. A total of 32 weaned rabbits, with an average live weight of 450 ± 20 g and approximately 56 days of age, were used in the study. The experiment included four treatments with eight replications each. The rabbits were housed individually in a controlled environment and subjected to the following experimental interventions: (1) A basal diet (control), (2) a basal diet supplemented with the commercial probiotic Biodep (0.02%), (3) a basal diet supplemented with the commercial prebiotic Agrimos (0.2%) and (4) a basal diet supplemented with a synbiotic, containing both the probiotic (0.02%) and prebiotic (0.2%). The study followed a completely randomized design. Feed rations were formulated according to the NRC standard (National Research Council 1977), as outlined by Halls (2010), and adjusted for the average age of the rabbits. The probiotic used contains strains of beneficial Gram‐positive bacteria, including species from the genera *Bacillus*, *Enterococcus*, *Lactobacillus*, *Pediococcus*, *Bifidobacterium* and *Streptococcus*. It may also include yeast and fungi, primarily strains of *S. cerevisiae*. The prebiotic consists of MOS and β‐glucan, derived from the cell wall of *S. cerevisiae*. It contains 36% protein, 50% carbohydrates and 3% crude fibre (CF).

### 2.2 Performance

To assess feed consumption, the leftover feed from the previous day was collected and weighed. The feed conversion ratio (FCR) was then calculated by dividing the average feed intake by the average live weight gain of the rabbits within each treatment group. Rabbit weights were recorded at the start of the study and every 14 days to evaluate fattening performance. This assessment included metrics such as daily weight gain, total feed consumption and the FCR (Table 1).

**TABLE 1:** Composition and calculated analyses (%) of the experimental diets on a dry matter basis.

		Treatment		
	Control	Probiotic (0.02%)	Prebiotic (0.2%)	Synbiotic^*^ (0.02% probiotic + 0.2% prebiotic)
**Feed ingredients %**				
Wheat bran	20.0	20.0	20.0	20.0
Alfalfa	30.0	30.0	30.0	30.0
Barley	35.0	35.0	35.0	35.0
Bagasse	5.0	5.0	5.0	5.0
Soybean meal	8.0	8.0	8.0	8.0
lime	1.0	1.0	1.0	1.0
NaCl	0.5	0.5	0.5	0.5
Vit‐Min. premix^b^	0.5	0.5	0.5	0.5
Calcium	0.90	0.90	0.90	0.90
Phosphors	0.54	0.54	0.54	0.54
**Chemical composition**				
Metabolism energy, KCal/kg	2400	2400	2400	2400
Crude protein	16.00	16.00	16.00	16.00
NDF	22.00	22.00	22.00	22.00
ADF	9.00	9.00	9.00	9.00
Dry matter	89.67	89.67	89.67	89.67
Ash	10.33	10.33	10.33	10.33

^*^Vitamin‐mineral mix: vitamin A, 10.000 IU; vitamin D3, 900 IU; vitamin E, 50 mg; vitamin K, 2 mg; vitamin B1, 2 mg; folic acid, 5 mg; pantothenic acid, 20 mg; vitamin B6, 2 mg; choline, 1.2 g; vitamin B12, 10 µg; niacin, 50 mg; biotin, 0.2 mg; Cu, 0.1 mg; Fe, 75 mg; Mn, 8.5 mg; Zn, 70 mg.

### 2.3 Digestibility

The measurement of the apparent digestibility of nutrients, including dry matter (DM), organic matter, crude fat, neutral detergent fibre (NDF) and crude protein (CP), was conducted using the ash method and acid‐insoluble ash technique. In this method, 5 g of feed or faeces were incinerated to ash and transferred to a 250‐mL Erlenmeyer flask. Then, 100 mL of 2N hydrochloric acid was added, and the mixture was boiled on a heater for 30 min. The resulting solution was filtered through the Whatman No. 41 filter paper. The walls of the Erlenmeyer flask were subsequently rinsed with hot distilled water, and the washings were also passed through the same filter paper. The filtrate was transferred to a pre‐weighed porcelain crucible, which was then placed in an oven at 600°C for 8 h. After cooling the crucible in a desiccator, its final weight was measured using a digital scale (Van Keulen and Young 1977).

The percentage of acid‐insoluble ash was calculated using the following equation:

*A* = weight of plant plus ash insoluble in acid (g), *B* = weight of the empty plant (g), *X* = weight of sample DM (g).

The following formula will be used to measure the digestibility of DM:

(1) $$\mathrm{Acid}\;\mathrm{in}\;\mathrm{insoluble}\;\mathrm{ash}\;\%\;=\frac{\left(A-B\right)\;\times \;100}{X}$$

(2) $$\begin{array}{ccc} & & \mathrm{Dry}\;\mathrm{material}\;\mathrm{apparent}\;\mathrm{digestibility}\%\;\\ & & \;=100-\left(100\times \frac{\mathrm{Food}\;\mathrm{in}\;\mathrm{AIA}\%}{\mathrm{In}\;\mathrm{faeces}\;\mathrm{AIA}\%}\right) \end{array}$$

(3) $$\begin{array}{ccc} & & \mathrm{Dry}\;\mathrm{material}\;\mathrm{apparent}\;\mathrm{digestibility}\%\;=100\\ & & -\left(100\times \frac{\mathrm{Food}\;\mathrm{in}\;\mathrm{AIA}\%}{\mathrm{In}\;\mathrm{faeces}\;\mathrm{AIA}\%}\times \frac{\mathrm{In}\;\mathrm{faeces}\;\mathrm{nourishment}\;\mathrm{of}\;\mathrm{matter}\%}{\mathrm{In}\;\mathrm{food}\;\mathrm{nourishment}\;\mathrm{of}\;\mathrm{matter}\%}\right) \end{array}$$

### 2.4 Blood Sampling and Analysis

For blood sampling, four rabbits were randomly selected from each treatment group. Fasting blood samples were collected and sent to Sina Pathology Laboratory Kits (13 Valiasr Adalat Street, Arman Complex, Gorgan, Iran) for analysis. The parameters measured included cholesterol, LDL, HDL, VLDL, triglycerides, glucose, white blood cells (lymphocytes, monocytes and granulocytes) and red blood cells (haemoglobin, haematocrit and platelets).

### 2.5 Skeletal Growth Trial

Skeletal growth in rabbits was assessed by measuring thigh length, arm length, chest circumference and total body length. These measurements were taken once at the beginning and once after the study period.

### 2.6 Intestinal Morphology

At the end of the study, four rabbits from each experimental group were slaughtered by severing the jugular veins of the neck using a sharp knife. Tissue samples from the duodenum, jejunum and ileum were collected and sent to the histology lab for evaluation of intestinal morphological changes. The histological indices were prepared as follows: intestinal tissue samples were fixed in 10% formalin and dehydrated using graded alcohol in ascending concentrations. The samples were then cleared with xylol. The processed tissue blocks were embedded in paraffin, and sections of 6 µm thickness were prepared using a LEICA RM 2145 rotary microtome. Before mounting, the sections were floated on 10% poly‐L‐lysine‐coated slides in warm water (55°C–60°C). The slides were stained with haematoxylin and eosin (Iji et al. 2001). Histological indices were analyzed using a computer‐aided optical cross‐image analyzer (Analysis Starter, Olympus, Japan). The height of the villi and the depth of the crypt were measured, and the villi height‐to‐crypt depth ratio was calculated. For analysis, the average values of 10 vertically aligned adjacent crypt units in each section were considered (Nwachukwu et al. 2021).

### 2.7 Statistical Analysis

The experiment was conducted based on a completely randomized design with four treatments and eight replications. Means were compared using Duncan's multiple range test with the help of SAS software version 9.1 according to the following models. *Yij = μ + Ti + eij*

In this regard, *Yij* is the value of each observation, *μ* is the average of observations, *Ti* is the effect of the experimental treatment and *eij* is the error of the experiment.

## 3 Results

### 3.1 Growth Performance

Table 2 presents the results for dry matter intake (DMI), initial weight, final body weight, average daily gain (ADG) and FCR. Body weight gain increased significantly (*p* < 0.05), and the FCR improved significantly (*p* < 0.05). During all measured periods, weeks 0–2, 2–4, 4–6, 6–8 and 0–8, the synbiotic diet group outperformed the probiotic, prebiotic and control groups in terms of daily weight gain and FCR. The probiotic and prebiotic groups showed similar results, whereas the control group demonstrated the lowest average daily weight gain.

**TABLE 2:** Effect of probiotics, prebiotics and synbiotics on growth performance (g/day).

Variables	Control	Probiotic (0.02%)	Prebiotic (0.2%)	Synbiotic	SEM	*p* value
Initial weight	675.3	650.9	658.5	653.5	−	−
Final weight	1315.0	1432.1	1383.6	1624.1	0.85	0.1300
Body weight gain, g						
0–2 weeks	8.82^d^	12.79^b^	12.09^c^	14.29^a^	0.25	< 0.0001
2–4 weeks	12.02^d^	14.08^b^	13.03^c^	18.01^a^	0.19	< 0.0001
4–6 weeks	12.35^d^	14.39^b^	13.06^c^	18.35^a^	0.21	< 0.0001
6–8 weeks	12.51^c^	14.54^b^	13.08^c^	18.69^a^	0.21	< 0.0001
0–8 weeks	11.42^d^	13.95^b^	12.95^c^	17.33^a^	0.11	< 0.0001
Feed intake, g						
0–2 weeks	78.17	77.44	77.75	76.98	0.58	0.5390
2–4 weeks	81.82	83.99	84.48	91.63	2.95	0.1250
4–6 weeks	90.01	90.50	92.00	94.55	2.25	0.4950
6–8 weeks	91.87	94.33	95.24	95.08	0.92	0.0550
0–8 weeks	85.47	86.56	87.37	89.56	1.02	0.0560
Total feed intake, g	4786.38	4847.88	4892.72	5015.59	57.57	0.0560
Feed conversion ratio (feed/gain)						
0–2 weeks	8.86^a^	6.05^c^	6.43^b^	5.38^d^	0.14	< 0.0001
2–4 weeks	6.81^a^	5.97^b^	6.48^a^	5.09^c^	0.23	< 0.0001
4–6 weeks	7.29^a^	6.29^b^	6.76^b^	5.15^c^	0.16	< 0.0001
6–8 weeks	7.34^a^	6.49^b^	7.35^c^	5.08^d^	0.13	< 0.0001
0–8 weeks	7.48^a^	6.20^b^	6.75^a^	5.16^c^	0.09	< 0.0001

*Note*: Means within a row bearing different superscript letters differ significantly (*p* < 0.05).

Abbreviation: SEM, standard error of means.

### 3.2 Nutrient Digestibility

The nutrient digestibility results for rabbits fed with probiotics, prebiotics and synbiotic supplements, including the digestibility of DM, organic matter, CP, crude fat and NDF, are presented in Table 3. The assessment revealed that the synbiotic treatment significantly enhanced the digestibility of DM, organic matter, crude fat and NDF (*p* ≤ 0.05).

**TABLE 3:** Effect of probiotics, prebiotics and synbiotics on nutrient digestibility (%).

Variables	Control	Probiotic	Prebiotic	Synbiotic	SEM	*p* value
Dry matter	64.82^c^	71.25^b^	69.29^b^	81.43^a^	0.99	< 0.0001
Organic matter	66.35^c^	72.39^b^	70.4^b^	82.40^a^	1.00	< 0.0001
Crude protein	89.32	85.78	84.47	87.31	3.11	0.726
Crude fat	75.55^b^	77.25^b^	78.47^b^	85.48^a^	1.21	< 0.0001
NDF	55.85^ab^	59.38^b^	51.22^c^	72.48^a^	1.89	< 0.0001

*Note*: Means within a row bearing different superscript letters differ significantly (*p* < 0.05).

Abbreviation: SEM, standard error of means.

### 3.3 Blood Parameters

The results of selected blood parameters for the experimental rabbits are presented in Table 4. A statistically significant difference was observed in cholesterol levels, white blood cell count, monocytes and mature neutrophils (Poly) among the experimental treatments, with the lowest levels recorded in the synbiotic group (*p* < 0.05). The remaining blood parameters showed no significant differences between the experimental and control groups.

**TABLE 4:** Haematological parameters of rabbits fed dietary probiotics and prebiotics diets (mg/dL).

Variables	Control	Probiotic	Prebiotic	Synbiotic	SEM	*p* value
Glucose, mg/dL	98.50	100.25	115.25	125.5	9.93	0.226
Triglyceride	137.5	109.75	137.0	105.5	15.18	0.325
Cholesterol	50.25^a^	31.00^b^	32.25^b^	31.25^b^	3.66	< 0.007
HDL	13.25	12.00	10.75	11.25	2.61	0.913
LDL	16.50	10.00	10.25	10.50	2.13	0.147
VLDL	27.50	21.95	27.40	21.10	3.03	0.325
WBC, ×10^3^/mL	21.76^a^	20.40^b^	21.49^a^	20.06^b^	7.03	< 0.002
RBC, ×10^6^/mL	5.475	5.322	5.585	5.175	0.18	0.446
Eosinophil	7.000	9.250	8.250	6.000	1.41	0.420
Monocyte	8.750^a^	7.500^a^	4.500^b^	4.250^b^	0.53	< 0.0001
Poly	70.75^a^	35.00^b^	44.50^b^	66.75^a^	4.04	< 0.0001

*Note*: Means within a row bearing different superscript letters differ significantly (*p* < 0.05).

Abbreviation: SEM, standard error of means.

### 3.4 Skeletal Growth

The performance results related to skeletal growth, hind leg bone length, hand bone length, chest length, waist length and abdominal girth are summarized in Table 5. A significant difference was found for all skeletal growth characteristics, except hind leg bone length (*p* < 0.05). The synbiotic supplement group exhibited the highest increase in bone growth, while the control group showed the lowest growth.

**TABLE 5:** Effects of probiotic, prebiotic and synbiotic supplements on bone growth in growing rabbits (cm).

	Variables	Control	Probiotic	Prebiotic	Synbiotic	SEM	*p* value
	Legs	16.62	16.77	16.93	16.78	0.18	0.694
Beginning	Hands	10.96	10.71	10.04	10.86	0.61	0.359
	Chest	12.85	12.42	12.75	13.06	0.21	0.226
	Waist	23.67	24.03	23.36	23.76	0.26	0.376
	Abdominal circumference	20.07^b^	20.78^ab^	21.52^a^	21.31^a^	0.27	< 0.004
	Legs	22.91	23.49	23.37	24.11	0.46	0.348
	Hands	16.09^c^	17.84^a^	16.88^b^	17.94^a^	0.19	< 0.0001
Day 56	Chest	16.69^b^	17.24^b^	17.17^b^	18.59^a^	0.36	< 0.0008
	Waist	32.08	33.70	32.68	35.00	0.78	< 0.067
	Abdominal circumference	23.26^d^	29.66^b^	28.13^c^	32.11^a^	0.43	< 0.0001

*Note*: Means within a row bearing different superscript letters differ significantly (*p* < 0.05).

Abbreviation: SEM, standard error of means.

### 3.5 Intestinal Morphology

**FIGURE 1:**
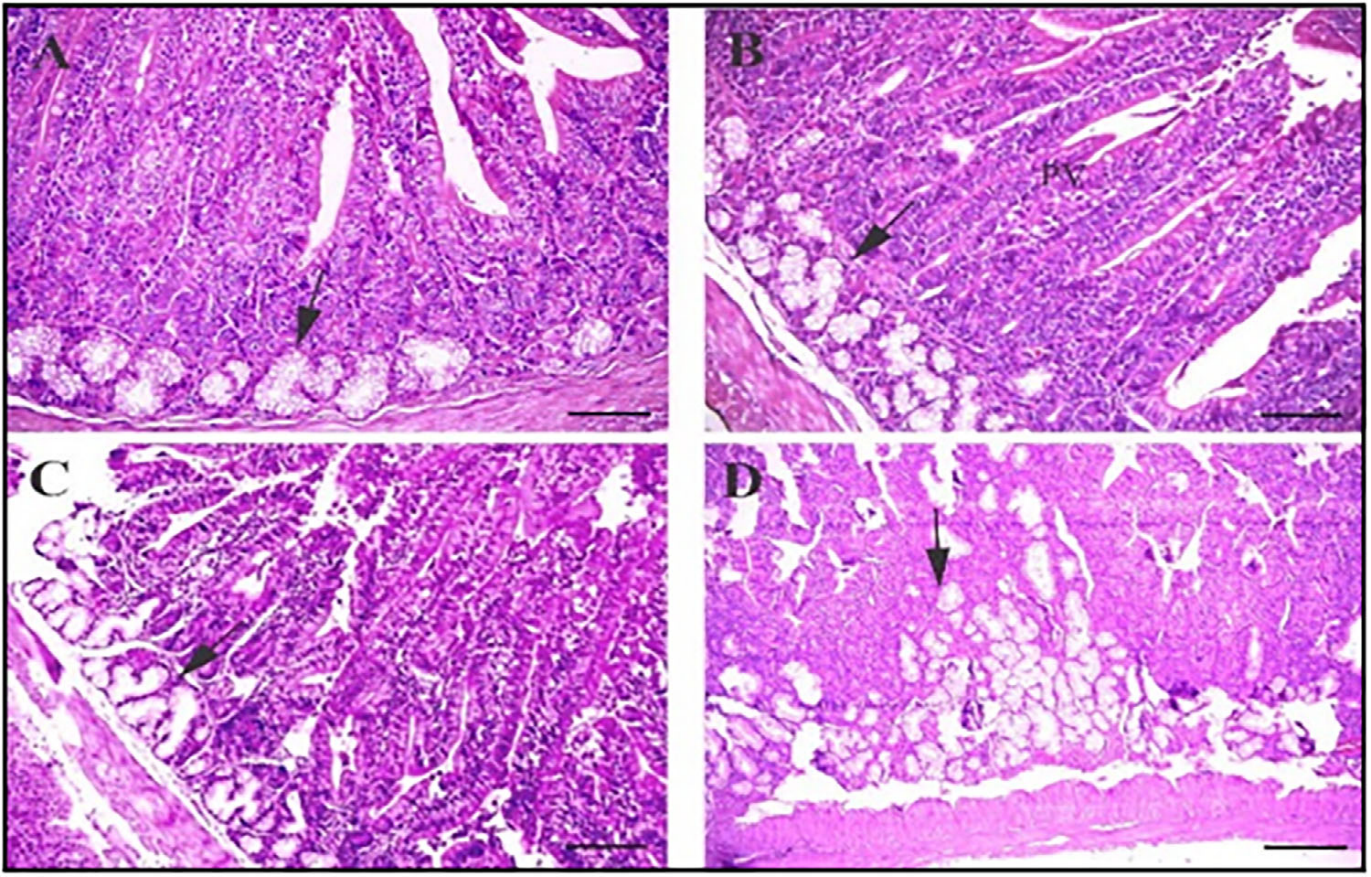
Histological sections of the duodenum from rabbits in different diets: arrows show the basal diet of Brunner's glands. Notably, Brunner's gland hypertrophy is observed in the symbiotic group. Scale bars = 100 µm. Staining with hematoxylin and eosin.

Notably, Brunner's gland hypertrophy is observed in the synbiotic group. Photomicrographs of enterocyte morphology in the duodenal villi of rabbits on various diets show distinct patterns. The duodenal villi in the prebiotic and probiotic groups exhibit a regular epithelial structure, as indicated by the arrows, whereas the control and symbiotic groups show slightly irregular epithelial morphology (Figure 1). Scale bars represent 50 µm, and sections were stained with haematoxylin and eosin.

## 4 Discussion

### 4.1 Feed Intake, Growth Performance and Daily FCR

In this study, significant differences were observed between the diet treatment groups and the control group. However, no significant differences in daily feed intake were detected among the diet treatments throughout the study period. An overall improvement trend was noted in the final live weight, daily weight gain and FCR across the treatment groups when compared to the control group. Nwachukwu et al. (2021) concluded that Biotronic probiotic growth promoters are safe for feeding rabbits, as they do not disrupt the activities of tissues, organs, or blood. These diets showed no oral toxicity but demonstrated beneficial effects on feed intake, absorption and utilization. Similarly, A. Abd El‐Aziz et al. (2020) investigated the effects of two dietary prebiotic oligosaccharide supplements on production performance and carcass traits in two breeds of rabbits, with a focus on biochemical changes. Their findings showed that the inclusion of prebiotic oligosaccharides, particularly MOS and isomalto‐oligosaccharide, significantly increased body weight and improved feed conversion efficiency. Studies utilizing synbiotics containing similar components demonstrated improvements in rabbit growth performance, albeit with varying effects on feed intake and FCRs. For instance, El‐Deeb et al. (2023) reported that feeding rabbits 0.5, 0.75 and 1.0 g/kg diets of synbiotics (containing β‐glucan, MOS and *Bacillus subtilis*) enhanced growth performance and feed intake, with the highest dose of 1.0 g/kg showing the most significant improvement, albeit without affecting FCRs. Similarly, Abo El‐Maaty et al. (2018) found that a diet supplemented with 0.5 g/kg of synbiotics containing MOS and a probiotic mix of *S. cerevisiae*, *Lactobacillus acidophilus*, *Streptococcus faecium* and other lactic acid bacteria resulted in comparable growth enhancements. In contrast, Hashem et al. (2021) reported enhanced growth performance and FCRs but no effect on feed intake when rabbits were fed encapsulated synbiotics (11 × 10^1^ ^1^ CFU of *S. cerevisiae* and 0.15 g Moringa oleifera leaf extract/kg diet). These findings align with broader research on other farm animals like poultry and pigs. Abdel‐Hamid and El‐Tarabany (2019) and de la Cruz et al. (2019) demonstrated significant improvements in weight gain and FCRs when incorporating similar feed additives. Çınar et al. (2009) observed positive effects on body weight and FCRs in broiler chickens fed with a combination of MOS and an antibiotic growth promoter (copper sulphate). Research consistently supports the notion that dietary supplements such as probiotics, prebiotics and organic acids and their combinations significantly improve body weight and growth performance across animal species. This aligns with the results of Çınar et al. (2009), who emphasized that prebiotics and synbiotics outperform probiotics in enhancing broiler chicken performance. Feeding of *Clostridium butyricum* in weaned rabbit was found to ameliorate weight gain through the probiotic activity on both digestive enzymes and small intestinal morphology (L. Liu et al. 2019). Further, in the digestive tract, the probiotic complex can multiply and synthetize itself a wide range of digestive enzymes while utilizing different carbohydrates to produce VFAs; thereby they not only extend the rabbit fermentative digestive system but also have a role in harmonizing the intestinal tract (L. Liu et al. 2019). Rabbit microbiota balance contributes to producing positive effects on productive performance and health. Indeed, mounting evidence suggested that probiotics effect on rabbit weight gain and feed utilization could be explained by the influence on gut health and microenvironment that in turn results in enhanced nutrient absorption capacity (Khalifa et al. 2024).

### 4.2 Digestibility of Nutrients

DM digestibility across the experimental treatments ranged from 64.82% to 81.43%. The highest DM digestibility was observed in the synbiotic treatment, while the probiotic and prebiotic treatments showed slightly lower but similar values. The lowest digestibility was recorded in the control group. The synbiotic treatment also demonstrated the highest organic matter digestibility, showing a significant difference compared to other treatments. Organic matter digestibility in the probiotic and prebiotic treatments was close, but both were significantly lower than that of the synbiotic treatment. The control group recorded the lowest organic matter digestibility at 66.35%, compared to 82.40% in the synbiotic group. CP digestibility ranged between 84.48% and 89.32% among the experimental treatments, with no significant differences observed between treatments. However, insoluble fibres in neutral detergent and crude fat digestibility showed significant differences across treatments. The synbiotic treatment yielded the highest digestibility for insoluble fibres in neutral detergent at 72.48%, whereas the prebiotic treatment recorded the lowest digestibility. Interestingly, the probiotic treatment's results for insoluble fibres were similar to those of the prebiotic group. The experimental treatments varied significantly for crude fat digestibility, ranging from 75.55% to 85.48%. The synbiotic treatment demonstrated the highest crude fat digestibility, while the control group exhibited the lowest. Probiotic‐fed rabbits demonstrated better absorption of vital nutrients. DM, organic matter, protein, fibre and energy were among these nutrients. Through the synergistic interaction of enzymatic activity, microbial fermentation, gut microbiota modulation and physiological adaptations, BS and LP improve digestibility traits in rabbits (Su et al. 2024). Complex proteins, fats and carbohydrates are broken down into absorbable monomers (like glucose, amino acids and fatty acids) by the amylases, lipases and proteases secreted by BS. Conversely, LP generates fibrolytic enzymes (such as hemicellulases and cellulases) that break down plant cell walls and liberate nutrients that have been trapped (Akinsemolu et al. 2024). Mountzouris et al. (2010) highlighted that the addition of probiotics, which contain a diverse pool of enzymes, may enhance nutrient digestibility in diets. Similarly, El‐Hindawy et al. (1993) observed that the inclusion of the probiotic Lacto‐Sacc improved the digestibility of all nutrients. Kamra et al. (2010) reported comparable improvements in CP digestibility, while Yamani et al. (2017) noted enhanced CF digestibility in rabbits. Amber et al. (2004) demonstrated that supplementation with *L. acidophilus* resulted in improved energy digestibility and analytical fractions, including CP, ether extract (EE) and DM, as well as CF digestibility. Bhatt et al. (2017) found that probiotic supplementation, specifically using *L. acidophilus* in concentrates, improved the digestibility of organic matter, CP and insoluble fibres in neutral detergent. They also reported significant improvements in body weight gain, FCR and carcass traits, including fatty acid composition, although carcass yield itself was unaffected. Moreover, optimizing gut microbial populations through probiotic supplementation enhances digestive efficiency, reduces feed costs and improves the utilization of fibre‐based feed. Similar observations have been made in other livestock species, demonstrating the broad potential of probiotics for improving nutrient utilization and performance.

### 4.3 Blood Biochemistry

The analysis of blood parameters in the experimental rabbits revealed a statistically significant difference in cholesterol levels, white blood cell count, monocytes and mature neutrophils (Poly) among the experimental treatments, with the lowest levels observed in the synbiotic group (*p* < 0.05). However, no significant differences were noted in the remaining blood parameters between the experimental and control groups. Khalid et al. (2011) reported that the high glucose concentration observed in rabbits consuming synbiotic supplements might result from increased bacterial activity within the supplement. This activity potentially converts carbohydrates into simpler substrates like glucose, which subsequently elevates blood glucose levels and provides the energy required for growth. Similarly, Etim et al. (2014) investigated the impact of nutrition on the haematology of rabbits, focusing on how dietary content influences their blood characteristics. Haematological studies serve as valuable tools for assessing the effects of various diets on the blood parameters of farm animals. These studies also explore the relationship between blood values and the animals' nutritional status. In general, as noted in previous research, the effects of probiotics, prebiotics and synbiotics on the biochemical and haematological parameters of rabbits vary significantly. While some studies found no notable impact of probiotics on these parameters, others observed significant increases or decreases in certain blood values. For example, Mancini and Paci (2021) highlighted these variations, underscoring the complexity of such nutritional interventions. Bhogoju and Nahashon (2022) confirmed that probiotics exhibit several predicted mechanisms, some of which are related to the inhibition of pathogenic intestinal microbes. A. N. Abd El‐Aziz et al. (2021) reported the use of yeast as a growth promoter in two breeds of growing rabbits, highlighting its economic implications. Specifically, supplementing rabbit diets with *S. cerevisiae* (at a rate of 0.12 g/kg diet) led to reduced blood cholesterol and total blood glycerides while increasing total blood protein, albumin and albumin/globulin ratios. In contrast, no significant differences were observed in total protein, albumin, globulin, albumin/globulin ratios, glucose, or triglyceride levels among groups supplemented with a probiotic mixture of *S. cerevisiae* and *Lactobacillus sporogenes* (at 0.5 and 0.1 g/kg diet); however, cholesterol levels decreased (Kalma et al. 2018). On the other hand, Mansour (2020) investigated the effects of the prebiotic Agri‐MOS feed on growth performance, carcass traits, blood plasma parameters, oxidative status and histomorphology in growing rabbits. The study revealed that adding MOS to rabbit diets at levels of 0.5, 0.75 and 1.0 g/kg significantly reduced serum glucose, triglycerides, LDL, aspartate aminotransferase, alanine aminotransferase and creatinine compared to the control group (Hassan et al. 2022). Research on immune indicators, growth performance, blood parameters, liver enzymes and kidney function in growing rabbits has examined the effects of bee pollen and/or MOSs. It was observed that MOS treatment (35 mg/kg body weight) led to a decrease in white blood cells, AST, ALT, urea and cholesterol concentrations, alongside an increase in glucose levels. However, no significant differences were recorded in total protein, triglycerides, or other haematological parameters. Regarding beta‐glucan supplementation, a dose of 10 mg/kg body weight increased total protein, globulin, white blood cells, lymphocytes and monocyte percentages in rabbits, while no effects were observed on albumin levels or the albumin/globulin ratio (Alkenany and Khalil, 2022). Additionally, Gabr (2021) reported that rabbits fed diets containing beta‐glucan at levels of 0.5, 1.0, 1.5 and 2.0 g/kg showed decreased glucose, triglyceride and total cholesterol concentrations across all levels, while no significant changes were observed in total protein, albumin, or globulin levels. Some researchers, such as Nami et al. (2019), have suggested that the inclusion of probiotics in animal feed reduces cholesterol levels through the direct absorption of cholesterol compounds by beneficial bacteria.

In addition, Mazhar et al. (2024a, 2024b) recorded that using probiotics improved the antioxidant capacity and immune system (indicated by a rise in serum globulin levels). The probiotic supplement stimulated the performance of growing rabbits and positively affected serum parameters, intestinal morphology, caecal ecology and microbiota (Tufarelli et al. 2025).

### 4.4 Skeletal Growth in Rabbits

The performance results related to bone growth in rabbits fed with probiotics, prebiotics and synbiotic supplements included measurements of hind leg bone length, forelimb bone length, chest length, waist length and abdominal girth. A significant difference was observed in all characteristics except for the hind leg bone length. The greatest increase in bone growth was recorded in the group receiving the synbiotic supplement, while the control group exhibited the lowest growth. Parvaneh et al. (2014) investigated the effects of probiotic supplements on bone mineral content and bone mass density, reporting positive outcomes in several animal studies and one human study. Most of the examined probiotics included *Lactobacillus* and *Bifidobacterium*. These positive effects were attributed to a high dietary calcium content and significant levels of supplemental probiotics. Key mechanisms identified include (1) increased solubility of minerals due to short‐chain fatty acid production. (2) Production of the phytase enzyme by bacteria to counteract the inhibitory effects of phytate on mineral absorption. (3) Reduction of intestinal inflammation, leading to improved bone mass density. (4) Hydrolysis of food glycosidic bonds in the intestine by *Lactobacillus* and *Bifidobacterium*. These mechanisms collectively enhance mineral bioavailability, demonstrating probiotics' potential in promoting bone metabolism through various pathways, with exceptional results observed in animal models. Sjögren et al. (2012) further supported this, identifying a mechanism where probiotic consumption reduced gut inflammation, consequently improving bone mass density. This emphasizes the significant role of gut microbes in bone health. Similarly, Kishawy et al. (2018) investigated the effects of dietary supplementation with whey powder and citric acid on the growth performance, nutrient digestibility, meat and bone analysis and gut health of growing rabbits. They found that this supplementation significantly increased bone calcium and phosphorus content, though it did not affect bone ash content. These findings suggest that citric acid's local accumulation in the bone matrix may facilitate co‐deposition during bone salt synthesis. Mohammed et al. (2021) studied the dietary supplementation of a probiotic (*B. subtilis*) on broiler chickens and reported that supplementation, particularly at a level of 0.5 g/kg, improved both bone mass and meat quality. This aligns with earlier studies, which established a direct correlation between probiotic feed additives and enhanced bone health and welfare in broiler chickens (Scholz‐Ahrens et al. 2007; McCabe et al. 2015). On the other hand, Rizzoli and Biver (2020) attributed the beneficial effects of probiotics on bone growth and strength to improved dietary digestibility and enhanced calcium and phosphorus absorption by beneficial intestinal bacteria. This increased the availability of serum calcium and phosphorus necessary for bone formation. Azad et al. (2025). *Bacillus subtilis* treatment significantly reduced the paw oedema and improved the arthritic index. The nocifensive threshold was also raised, and muscle coordination improved considerably after *B. subtilis* treatment. The antioxidant capacity and histological and radiological parameters were also enhanced Khan et al. (2025). An in vitro safety evaluation of new plant lactic acid bacteria isolated from fruits as potential probiotics concluded that fresh fruits are a good source of lactic acid bacteria. These bacteria may be candidates for plant probiotics in the future. Enterococcus strains isolated from local fruits are non‐haemolytic and DNase negative. These strains also have antioxidant properties and antibiotic susceptibility.

### 4.5 Intestinal Morphology

**FIGURE 2:**
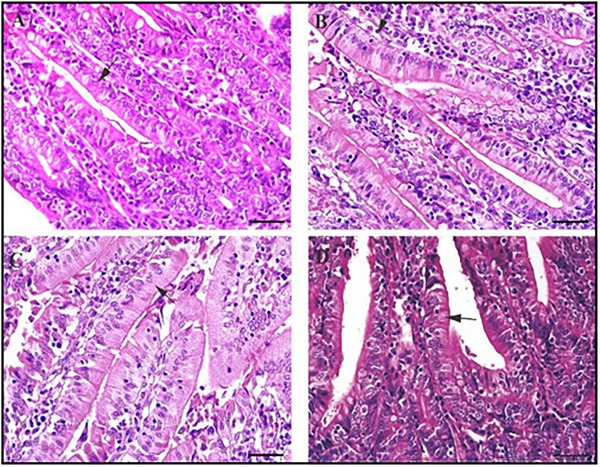
Photomicrographs of enterocyte morphology in the duodenal villi of rabbits on various diets the duodenal villi in the prebiotic and probiotic groups exhibit a regular epithelial structure (arrows), whereas the control and symbiotic groups.

The intestinal morphology results of rabbits fed with probiotic, prebiotic and synbiotic supplements are shown in Figure 2. Histological sections of the duodenum from rabbits on different diets highlight the basal diet's effects on Brunner's glands, as indicated by the arrows. Notably, Brunner's gland hypertrophy is observed in the synbiotic group. Scale bars represent 100 µm. Sections were stained with haematoxylin and eosin for detailed visualization. Intestinal villi and crypt morphometric measurements are crucial markers of digestive health. Since crypt cells help to renew the intestinal wall's lining absorptive epithelial cells, a rise in VH may be exactly associated with the expansion of absorptive surface area and cell proliferation (Tufarelli et al. 2025), whereas the CD rate might be associated with the degree of cell turnover. Accordingly, a higher VH: CD ratio denotes a greater potential for absorbing nutrients (Rafieian‐Naeini et al. 2024). In addition to improving digestion and nutritional absorption, the longer villi and larger surface area may also increase the expression and activity of brush border enzymes. Mourão et al. (2006) reported that the crypt width in the duodenum was highest in the control group, with no significant differences observed in the jejunum or ileum. Similarly, Awad et al. (2009) noted an increase in villus height and the villus‐to‐crypt depth ratio in the duodenum and ileum, suggesting enhanced epithelial cell turnover. However, Oso et al. (2013) found no improvement in intestinal morphology after administering *Pediococcus acidilactis* and *Bacillus cereus* to weanling rabbits.

## 5 Conclusion

Based on the findings of this study, synbiotics demonstrated significant improvements in growth performance, feed utilization and overall rabbit health. They also enhanced skeletal growth and reduced cholesterol levels in comparison to prebiotics or probiotics used individually. Consequently, synbiotics may represent a promising nutrient choice for the future. The synergistic effects observed with the current synbiotic formulation highlight its potential to contribute to sustainable rabbit production and reduce reliance on antibiotics. This makes synbiotics a recommended option for use in rabbit breeding under commercial production conditions. However, further research is needed to better understand the regulatory role of synbiotics on gut health and to elucidate their mechanisms of action.

## Author Contributions

**Khalaf Alrajab**: conceptualization, investigation, methodology, validation, visualization, writing – review and editing, project administration, funding acquisition, writing – original draft. **Taghi Ghoorchi**: conceptualization, investigation, methodology, validation, software, formal analysis, data curation, supervision, funding acquisition, writing – original draft, project administration, resources. **Abdolhakim Toghdory**: investigation, conceptualization, validation, formal analysis, supervision, visualization, resources. **Omid Ashayerizade**: conceptualization, investigation, validation, software, supervision, data curation. **Farzaneh Ganji**: conceptualization, investigation, validation, formal analysis, supervision.

## Funding

The authors have nothing to report.

## Disclosure

We certify that the submission is original work and is not under review at any other publication. We confirm that the article we have submitted has been prepared in my personal capacity and not as an official representative or on behalf of any sanctioned government.

## Ethics Statement

All experimental procedures involving animals were conducted in accordance with the ethical standards for animal experimentation and complied with the guidelines for the care and use of laboratory animals. All efforts were made to minimize animal suffering and to reduce the number of animals used. Rabbits were housed under appropriate environmental and hygienic conditions throughout the experimental period. Slaughtering procedures were performed humanely by severing the jugular vein using a sharp knife to ensure rapid exsanguination and minimal distress, in accordance with accepted animal welfare.

## Conflicts of Interest

The authors declare no conflicts of interest.

## Data Availability

The data that support the findings of this study are available from the corresponding author.
